# From the court to the heart: basketball intervention is associated with adolescents’ subjective well-being—a serial mediation analysis of executive function and psychological capital

**DOI:** 10.3389/fpsyg.2026.1929704

**Published:** 2026-08-10

**Authors:** Donghai Huang, Yan Li, Pengyun Zhang, Gang Zhao, Hang Zhang, Ming Zhang, Jianping Xiong, Muhammad Nubli Abdul Wahab, Heng Wang

**Affiliations:** 1 School of Physical Education, Ningxia Normal University, Guyuan, China; 2 College of Physical Education, Henan Normal University, Xinxiang, China; 3 Faculty of Education, Henan Normal University, Xinxiang, China; 4 Center for Human Science, University Malaysia Pahang Al-Sultan Abdullah, Pekan, Pahang, Malaysia

**Keywords:** basketball intervention, executive function, psychological capital, serial mediation, subjective well-being

## Abstract

**Aim:**

This study aims to elucidate the psychological mechanisms through which basketball intervention is associated with adolescents’ subjective well-being (SWB).

**Methods:**

A 2 × 2 mixed experimental design was employed, and 64 high school students were randomly assigned to an experimental group (n = 32) and a control group (n = 32). The experimental group received a 12-week moderate-intensity basketball intervention conducted three times per week for 30 min per session, whereas the control group participated only in regular physical education classes. Executive function (EF), psychological capital (PsyCap), and SWB were measured at baseline and 1 week post-intervention in both groups. Mediation analysis was conducted using the PROCESS macro in SPSS.

**Results:**

The results indicated that basketball intervention was significantly associated with higher adolescents’ SWB (total effect: β = 0.734; direct effect: β = 0.255; indirect effect: β = 0.479). EF (inhibitory control: β = 0.204; updating: β = 0.232; cognitive flexibility: β = 0.172) and PsyCap (β = 0.233) emerged as statistical partial mediators, forming a serial mediation pathway linking executive function and PsyCap.

**Conclusion:**

Basketball intervention showed positive direct and indirect associations with adolescents’ SWB, which were linked to the combined roles of cognitive and psychological resources.

## 1 Introduction

Subjective well-being (SWB) is the overall evaluation of an individual’s life based on subjective experiences, encompassing cognitive judgments of life satisfaction and affective experiences of positive and negative emotions (Martínez-Líbano et al., 2025). Recent data shows a consistent decline in adolescents’ SWB. The Guidelines on Measuring SWB indicated that around 18% of adolescents reported low life satisfaction in 2025, up from 12% in 2015 (Organisation for Economic Co-operation and Development, 2025). In China, approximately 15%–25% of adolescents are at risk of mental health issues, with heightened negative emotions (Institute of Psychology, Chinese Academy of Sciences, 2025). Studies have shown that prolonged exposure to low SWB not only affects emotional regulation and psychological adjustment in adolescents, but also impacts their physical and mental health development (Matić and Musil, 2023). Therefore, identifying effective intervention strategies to improve adolescents’ SWB and understanding the underlying mechanisms are crucial in adolescent education and mental health research.

Among the various intervention strategies available, exercise intervention has increasingly been recognized as an important approach for enhancing adolescents’ SWB due to its low cost, high feasibility, and positive psychological effects (Ruiz-Ranz and Asín-Izquierdo, 2025). A substantial body of empirical studies and meta-analytic evidence has demonstrated that participation in structured exercise programs can significantly improve individuals’ SWB, life satisfaction, and positive emotional experiences (Cao et al., 2025). However, existing research has primarily focused on general physical activity or broader exercise programs (Penedo and Dahn, 2005), while relatively little attention has been paid to basketball as a structured form of exercise that integrates physical activity, social interaction, and team collaboration. Compared with traditional individual sports, basketball not only requires continuous physical participation but also demands team coordination, role collaboration, and real-time decision-making in dynamic situations (Kong et al., 2026; Iván-Baragaño et al., 2025). Such a highly interactive sporting environment may provide individuals with richer positive emotional experiences, a stronger sense of belonging, and more opportunities for success experiences (Wilkie et al., 2026), thereby potentially facilitating the accumulation of psychological resources and the enhancement of SWB. Based on the above evidence, this study proposes:

Hypothesis 1: Basketball intervention will be significantly associated with higher adolescents’ SWB.

Executive function (EF) is a core cognitive ability essential for goal-directed behavior, self-regulation, and cognitive control. It significantly influences emotion regulation and SWB (Hofmann et al., 2012). Because EF depends on sustained cognitive engagement and behavioral regulation, exercise environments can support its development (Bigliassi et al., 2026) Research shows that regular exercise improves EF, especially inhibitory control, working memory updating, and cognitive flexibility (Liu et al., 2025). Structured team-based exercise programs may have a stronger effect on EF due to greater demands on decision-making, attentional shifting, and behavioral control (Cabral et al., 2025). Basketball, as a typical open-skill and competitive team sport, requires continuous attention, rapid decisions, ongoing information processing, and behavioral regulation in dynamic situations (Alemanno et al., 2025). Moreover, EF development is closely linked to adolescents’ SWB. Higher EF supports better emotion regulation, stress management, and goal achievement, which reduce negative emotions and enhance positive psychological states (Diamond and Ling, 2016). Evidence also suggests that individuals with stronger EF tend to have improved psychological well-being and greater life satisfaction (Cabral et al., 2025). Together, these findings lead to:

Hypothesis 2: EF serves as a mediator in the relationship between basketball intervention and adolescents’ SWB.

Beyond cognitive regulatory capacities, positive psychological resources may also play an important role in the relationship between basketball participation and adolescents’ SWB. Psychological capital (PsyCap), as a form of positive psychological resource, primarily comprises four core components: self-efficacy, hope, optimism, and resilience (Goel and Wani, 2024). Empirical evidence indicates that higher levels of PsyCap can enhance positive emotional experiences and life satisfaction, thereby contributing to higher levels of SWB (Zhou et al., 2025). Among adolescents, individuals with higher levels of PsyCap tend to develop more positive self-evaluations and future expectations and maintain greater emotional stability and higher SWB when facing academic stress and social adaptation challenges (Sun, 2025). Previous research has found that participation in exercise contributes to the enhancement of self-efficacy and hope by providing clear goals, continuous feedback, and opportunities for successful experiences (Luo et al., 2025). Regular physical activity can also enhance self-efficacy, alleviate stress, and improve psychological adjustment, thereby fostering the development of positive psychological traits such as optimism and resilience (Zhou et al., 2025). Compared with general physical activity, exercise settings characterized by stronger team interaction may provide richer opportunities for positive feedback, peer encouragement, and successful experiences, thereby facilitating the development of positive psychological resources. Basketball participation may further enhance positive psychological experiences, including a stronger sense of belonging, greater optimism, and higher levels of hope (Zhang and Siriphan, 2024). During basketball participation, adolescents are more likely to develop positive psychological resources and favorable self-evaluations through team cooperation, role involvement, and successful experiences (Wang, 2026). Accordingly:

Hypothesis 3: Psychological capital serves as a mediator in the relationship between basketball intervention and adolescents’ SWB.

Existing evidence suggests that EF plays a critical role in cognitive regulation and is also considered an important cognitive foundation for the development of positive psychological resources (Strömbäck et al., 2020). Higher levels of EF help individuals maintain positive expectations and stable psychological states when facing stress and challenges (Lenartowicz et al., 2021). Previous studies have suggested that improvements in EF may facilitate the formation of positive psychological resources and provide important cognitive support for the development of PsyCap (Diamond and Ling, 2016). From the perspective of cognitive resource theory (Bruya and Tang, 2018), individuals with stronger EF possess greater cognitive capacity to allocate attentional and regulatory resources toward positive goal pursuit, a process that serves as the foundation for self-efficacy, hope, optimism, and resilience. Specifically, strong inhibitory control helps individuals suppress maladaptive negative thoughts, thereby enhancing optimism and hope (Bishara and Kaplan, 2022); cognitive flexibility enables individuals to reinterpret setbacks and maintain self-efficacy (Sun, 2025); and working memory updating facilitates the retention of goal-relevant positive information, fostering resilience (Strömbäck et al., 2020). Therefore, this study simultaneously incorporates EF and PsyCap into the relationship between basketball intervention and SWB to examine the interrelationships among these variables and to explore the mechanisms through which basketball intervention influences adolescents’ SWB. Within the context of sport participation, basketball provides a rich environment for cognitive regulation and the development of positive psychological resources due to its high-intensity intermittent nature, continuous cognitive engagement demands, and socially interactive competitive structure. On the one hand, frequent decision-making, attentional shifting, and behavioral inhibition during basketball participation help strengthen EF and enhance self-regulatory capacity (Xue et al., 2019). On the other hand, the positive feedback derived from teamwork, role differentiation, and successful experiences can further enhance individuals’ self-efficacy, optimism, and resilience (Morgan, 2018). Therefore, basketball participation may be associated with adolescents’ SWB through its relationships with EF and PsyCap, potentially forming a sequential indirect pathway of influence. Building on the foregoing arguments:

Hypothesis 4: EF and PsyCap serve as sequential mediators in the relationship between basketball intervention and adolescents’ SWB.

**Figure 1:**
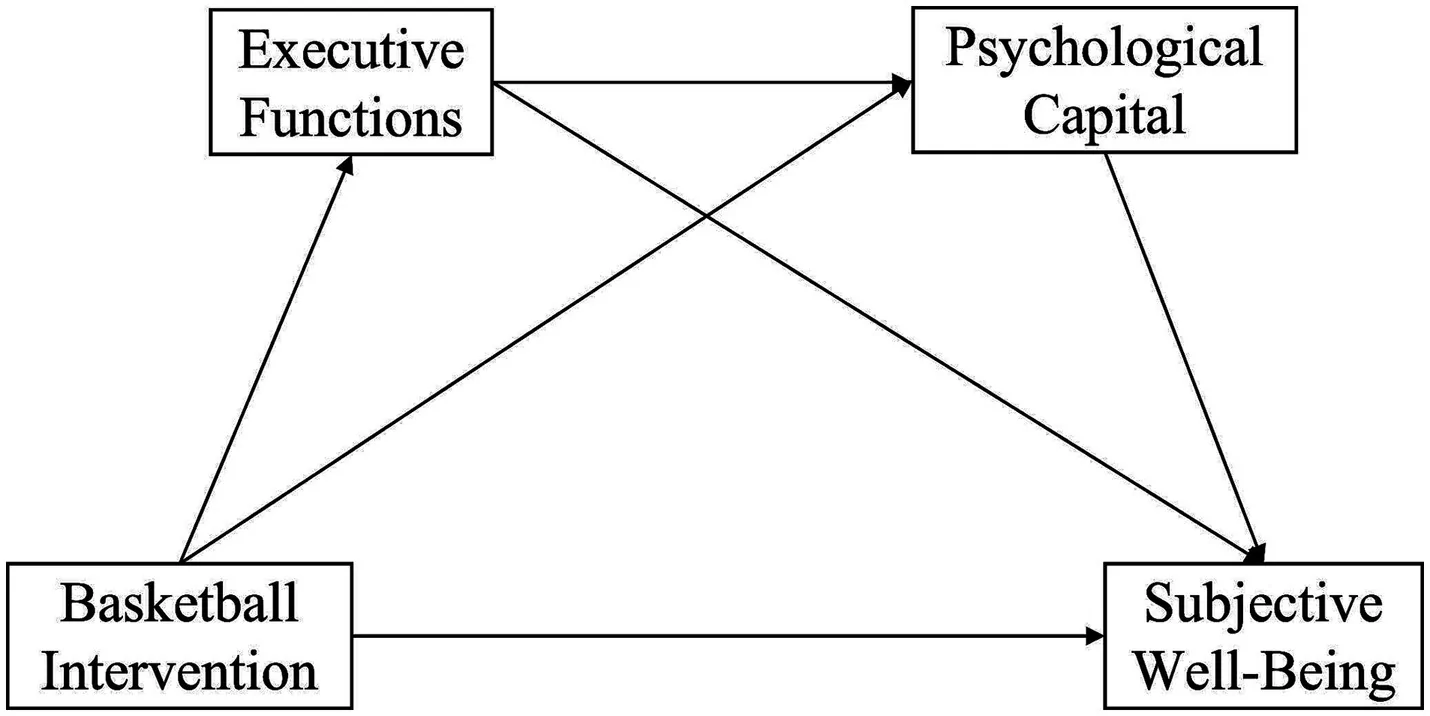
Research hypothesis model.

In summary, this study proposes a serial mediation model (Figure 1) to examine the associations of basketball intervention on adolescents’ SWB and to investigate the independent and sequential the independent and sequential mediating pathways involving EF and PsyCap. Specifically, we aim to elucidate the pattern of relationships among EF, PsyCap, and SWB following the basketball intervention, rather than to estimate causal effects of change scores.

## 2 Methods

### 2.1 Participants

Participants were recruited via campus bulletin boards and posters at a public high school in China. Recruitment was conducted for a school basketball program. A total of 137 students were recruited. After screening, 64 participants met the inclusion criteria. The inclusion criteria were as follows: (a) Physically healthy, without prior systematic training, and able to participate in regular physical activity, with a commitment to follow the study protocol; (b) Normal cognitive functioning was confirmed during recruitment through school enrollment records and teacher verification (see Supplementary methods for details); (c) No color blindness or color weakness; (d) Provided informed consent. Sample size estimation was conducted using G*Power 3.1.9.6. The significance level was set at *α* = 0.05, the effect size at *f* = 0.25, and statistical power at 0.80 (1 − *β*). The required minimum sample size was calculated to be 34 participants.

### 2.2 Design

A total of 64 participants were randomly assigned to an experimental group (*n* = 32) and a control group (*n* = 32). The random allocation sequence was generated by the first author (DH) using the random number function in Microsoft Excel. A research assistant (HZ) who was not involved in participant recruitment or outcome assessment assigned participants to the groups according to the sequence. Allocation concealment was ensured by using sequentially numbered, sealed, opaque envelopes, which were opened only after the baseline assessment was completed. A partial blinding design was adopted. Due to the nature of the physical activity intervention, participants and the physical education teacher could not be blinded. The outcome assessors responsible for post-test data collection and scoring (MZ and GZ) were blinded to group allocation. The purpose of assessor blinding was to reduce potential measurement bias during the post-test assessment. In addition, data analysis was performed by a statistician (JX) who was not involved in data collection and remained blinded to group assignment throughout the analysis.

The experimental group participated in a 12-week basketball intervention, conducted three times per week, with each session lasting 30 min (excluding warm-up and cool-down). The intervention was performed at a moderate intensity. Exercise intensity was determined based on the guidelines of the American College of Sports Medicine (ACSM). Moderate intensity was defined as 60%–69% of the age-predicted maximum heart rate, calculated as (220 − age) × 60%–69%. Participants wore Polar heart rate monitors (Polar Electro, Finland) during each session to ensure adherence to the target intensity. During the 12-week intervention, attendance was recorded by the physical education teacher at each session. The average attendance rate in the experimental group was 94.4% (34 out of 36 sessions). The average percentage of training time spent within the target heart rate zone (60%–69% of age-predicted maximum heart rate) was 79.4%, indicating satisfactory compliance with the prescribed intensity. No adverse events were reported during the study. The intervention program (Supplementary Table S1, S2) was designed based on the Chinese Physical Education and Health Curriculum Standards and related instructional materials. It included dribbling, running dribbling, passing and receiving, stationary one-hand shoulder shooting, running one-hand shoulder shooting, tactical training, and game-based practice. The control group maintained their usual daily routines and school activities. They did not receive any additional exercise intervention beyond regular physical education classes. Measurements of SWB, EF, and PsyCap were conducted at baseline and after the intervention for both groups. Participants who engaged in other structured exercise training during the study were excluded from the analysis.

### 2.3 Measures

#### 2.3.1 Subjective well-being

Subjective Well-Being was assessed using the Adolescent SWB Scale developed by Zhang (2003). The scale consists of 36 items and covers six dimensions: family, friendship, academics, school, freedom, and environment. It provides a comprehensive assessment of adolescents’ perceived quality of life. All items were rated on a 7-point Likert scale ranging from 1 (strongly disagree) to 7 (strongly agree). Items 3, 4, 9, and 10 were reverse-coded. Scores for each dimension were calculated as the sum of the corresponding items. The overall SWB score was computed as the sum of the six dimensions. Higher scores indicate higher levels of SWB. In the present study, the scale demonstrated high internal consistency, with a Cronbach’s *α* of 0.911.

#### 2.3.2 Psychological capital

Psychological capital was assessed using the Positive PsyCap Questionnaire (PPQ) developed by Zhang et al. (2010). The questionnaire consists of 26 items and includes four dimensions: self-efficacy, resilience, hope, and optimism. All items were rated on a 7-point Likert scale ranging from 1 (strongly disagree) to 7 (strongly agree). Items 8, 10, 12, 14, and 25 were reverse-coded. Scores for each dimension were calculated as the sum of the corresponding items. The overall PsyCap score was computed as the sum of the four dimensions. Higher scores indicate higher levels of PsyCap. In the present study, the questionnaire demonstrated good internal consistency, with a Cronbach’s α of 0.892.

#### 2.3.3 Executive function

Executive function tasks were administered on a laptop computer using E-Prime 3.0 software. All participants received standardized instructions and were required to achieve an accuracy rate of at least 80% in the practice trials before proceeding to the formal test. This criterion was applied only to the practice trials, and all participants met this criterion (no exclusions). The average accuracy rates for the three tasks were 80.6% (Flanker), 81.3% (2-back), and 84.5% (More-odd shifting). The tasks were administered in the following order: Flanker, 2-back, and More-odd shifting, with a 5-min rest between tasks. Detailed task parameters (trial numbers, stimulus characteristics, timing, response procedures, and trial condition proportions) are provided in Supplementary Table S3.

Inhibitory control was assessed using the Flanker task (Eriksen and Eriksen, 1974). The outcome measure was the conflict effect (incongruent trial mean RT minus congruent trial mean RT), with smaller values indicating better inhibitory control. Working memory updating was assessed using the 2-back task (Smith and Jonides, 1997). The outcome measure was the mean RT for correct responses, with shorter values indicating better updating ability. Cognitive flexibility was assessed using the More-odd shifting task (Salthouse et al., 2003). The outcome measure was the switch cost (switch trial mean RT minus non-switch trial mean RT), with smaller values indicating better cognitive flexibility. These difference-based measures were selected because they isolate the specific EF component of interest while controlling for general processing speed (Miyake et al., 2000). For all tasks, trials with reaction times below 200 ms or above 2,000 ms were excluded as implausible responses (affecting <2% of trials).

### 2.4 Data analysis

All statistical analyses were conducted using SPSS 29.0. First, independent samples t-tests were performed to examine whether the experimental and control groups were equivalent at baseline in SWB, PsyCap, and EF. Second, repeated-measures analysis of variance (RM-ANOVA) was used to examine the effects of the basketball intervention. For significant interaction effects, simple effects analyses were conducted. Third, Pearson correlation analysis was performed to examine the relationships among variables. Fourth, multiple linear regression diagnostics were conducted to assess multicollinearity among variables and the independence of residuals was supported by the Durbin-Watson test. Fifth, mediation analyses were conducted using the PROCESS macro (Model 6) in SPSS to test the serial mediation model. Post-test scores of EF, PsyCap, and SWB were entered as the mediator and outcome variables. All variables were standardized prior to analysis. Sixth, Bootstrap resampling (5,000 samples) was used to estimate the significance and robustness of the indirect pathways.

## 3 Results

### 3.1 Subsection baseline equivalence

All 64 randomized participants completed the post-test assessment (100% retention). Before the intervention, no significant differences were observed between the experimental and control groups in SWB, EF, or PsyCap (*p* > 0.05), indicating good baseline equivalence between the two groups (Supplementary Table S4).

### 3.2 Effects of basketball intervention on adolescents’ subjective well-being, executive function, and psychological capital

The repeated-measures ANOVA revealed significant Time × Group interaction effects for inhibitory control (Table 1), *F* (1, 62) = 7.490, *p* = 0.008, η_p_^2^ = 0.108; working memory updating, *F* (1, 62) = 10.200, *p* = 0.002, η_p_^2^ = 0.141; and cognitive flexibility, *F* (1, 62) = 4.123, *p* = 0.047, η_p_^2^ = 0.062 following the 12-week basketball intervention. Within-group comparisons indicated that EF reaction times were significantly lower at post-test than at pre-test in the experimental group, whereas no significant changes were observed in the control group. These findings suggest that basketball intervention was associated with higher aspects of adolescents’ EF, with the strongest associations observed for inhibitory control and working memory updating, and a marginal association for cognitive flexibility.

**Table 1:** Time × group interaction effects of basketball intervention on adolescents’ subjective well-being, psychological capital, and executive function.

Dimension	Experimental group		Control group		Time		Group		Time × Group	
	Pre-test	Post-test	Pre-test	Post-test	*F*	$\eta_{p}^{2}$	*F*	$\eta_{p}^{2}$	*F*	$\eta_{p}^{2}$
SWB										
Family satisfaction	35.38 ± 3.62	36.59 ± 4.29	34.88 ± 4.50	34.97 ± 4.01	3.403	0.052	1.261	0.020	2.500	0.292
Academic satisfaction	24.38 ± 6.53	25.53 ± 4.27	23.09 ± 6.27	23.44 ± 3.37	6.260	0.092	4.709	0.071	0.603	0.010
School satisfaction	26.84 ± 6.65	32.19 ± 3.85	25.31 ± 4.48	26.56 ± 4.23	39.670^***^	0.390	10.300^**^	0.142	15.291^***^	0.198
Environmental satisfaction	24.78 ± 4.58	30.47 ± 2.01	23.56 ± 5.18	24.94 ± 2.53	33.651^***^	0.352	20.964^***^	0.253	12.547^***^	0.168
Freedom satisfaction	24.72 ± 4.00	28.87 ± 3.25	23.19 ± 3.68	24.19 ± 3.21	24.281^***^	0.281	18.831^***^	0.233	9.098^**^	0.128
Friendship satisfaction	39.47 ± 5.16	45.09 ± 3.85	37.31 ± 5.43	38.94 ± 4.33	39.591^***^	0.390	16.190^***^	0.207	12.052^***^	0.163
Total SWB	175.22 ± 20.07	198.59 ± 16.43	174.91 ± 12.07	178.06 ± 7.38	62.549^***^	0.502	12.601^***^	0.169	36.325^***^	0.369
PsyCap										
Self-efficacy	24.88 ± 6.19	28.97 ± 4.36	23.09 ± 7.05	24.75 ± 5.35	41.625^***^	0.402	4.685^*^	0.070	7.480^**^	0.108
Resilience	27.19 ± 5.08	29.53 ± 3.13	25.75 ± 4.64	26.87 ± 2.67	5.395^*^	0.080	8.987^**^	0.127	4.357^*^	0.066
Optimism	25.66 ± 4.85	30.59 ± 5.10	23.97 ± 4.40	25.69 ± 1.89	33.338^***^	0.350	13.600^***^	0.180	7.796^**^	0.112
Hope	24.19 ± 4.61	30.06 ± 3.58	23.91 ± 4.36	25.19 ± 2.98	42.620^***^	0.407	9.957^**^	0.138	17.562^***^	0.221
Total PsyCap	100.78 ± 17.22	119.16 ± 19.96	98.94 ± 11.08	101.5 ± 9.15	70.329^***^	0.531	11.622^***^	0.158	40.113^***^	0.393
EF										
Inhibitory control	30.01 ± 9.93	24.88 ± 7.01	32.20 ± 6.56	30.28 ± 3.53	15.408^***^	0.199	7.149^*^	0.103	7.490^**^	0.108
Working memory updating	990.61 ± 159.78	817.77 ± 77	971.22 ± 171.51	946.23 ± 101.17	18.259^**^	0.228	5.168^*^	0.077	10.200^**^	0.141
Cognitive flexibility	550.18 ± 94.43	494.32 ± 39.32	579.96 ± 88.48	570.14 ± 78.03	8.384^**^	0.110	11.051^***^	0.151	4.123^*^	0.062

SWB, subjective well-Being; PsyCap, Psychological Capital; EF, executive function; ***p < 0.001, **p < 0.01, *p < 0.05.

For psychological capital, significant Time × Group interaction effects were observed for self-efficacy, *F* (1, 62) = 7.480, *p* < 0.01, η_p_^2^ = 0.108; resilience, *F* (1, 62) = 4.357, *p* < 0.05, η_p_^2^ = 0.066; optimism, *F* (1, 62) = 7.796, *p* < 0.01, η_p_^2^ = 0.122; hope, *F* (1, 62) = 17.562, *p* < 0.001, η_p_^2^ = 0.221; and overall PsyCap *F* (1, 62) = 40.113, *p* < 0.001, η_p_^2^ = 0.393. Further analyses indicated that PsyCap scores in the experimental group were significantly higher at post-test than at pre-test, whereas no significant changes were found in the control group, suggesting that basketball intervention effectively promoted the development of PsyCap among adolescents.

Regarding subjective well-being, significant Time × Group interaction effects were found for school satisfaction, *F* (1, 62) = 15.291, *p* < 0.001, η_p_^2^ = 0.198; environmental satisfaction, *F* (1, 62) = 12.547, *p* < 0.001, η_p_^2^ = 0.168; freedom satisfaction, *F* (1, 62) = 9.098, *p* < 0.01, η_p_^2^ = 0.128; friendship satisfaction, *F* (1, 62) = 12.052, *p* < 0.001, η_p_^2^ = 0.163; and overall SWB, *F* (1, 62) = 36.325, *p* < 0.001, η_p_^2^ = 0.369. These findings indicate that basketball intervention was significantly associated with higher adolescents’ SWB. In contrast, no significant interaction effects were observed for family satisfaction or academic satisfaction.

### 3.3 Correlation analysis among variables

Pearson correlation analysis was conducted to examine the relationships among the variables (Table 2). The results showed that basketball intervention was significantly positively correlated with SWB and PsyCap (*p* < 0.01), and significantly negatively correlated with inhibitory control, working memory updating, and cognitive flexibility (*p* < 0.01). SWB was significantly negatively correlated with inhibitory control, cognitive flexibility, and working memory updating (*p* < 0.01), and significantly positively correlated with PsyCap (*p* < 0.01). Inhibitory control, cognitive flexibility, and working memory updating were significantly negatively correlated with PsyCap (*p* < 0.01).

**Table 2:** Correlations among basketball intervention, subjective well-being, executive function, and psychological capital.

	1. Basketball intervention	2. SWB	3. PsyCap	4. Inhibitory control	5. Cognitive flexibility	6. Working memory updating
1. Basketball intervention	—					
2. SWB	0.734^**^	—				
3. PsyCap	0.664^**^	0.818^**^	—			
4. Inhibitory control	−0.506^**^	−0.786^**^	−0.650^**^	—		
5. Cognitive flexibility	−0.529^**^	−0.723^**^	−0.570^**^	0.482^**^	—	
6. Working memory updating	−0.587^**^	−0.804^**^	−0.694^**^	0.629^**^	0.705^**^	—

SWB, Subjective Well-Being; PsyCap, Psychological Capital. *p < 0.05, **p < 0.01, ***p < 0.001.

### 3.4 Multicollinearity diagnostics

Prior to the mediation analyses, the assumptions underlying the serial mediation model were assessed. The linearity of the relationships among variables was plausible based on visual inspection of partial regression plots. The independence of residuals was supported by the Durbin-Watson test (values = 1.853), indicating no significant autocorrelation. Given that the serial mediation analyses were conducted using the PROCESS macro with 5,000 bootstrap samples, the assumptions of normality and homoscedasticity were not required (Hayes, 2017). In addition, to prevent potential multicollinearity issues arising from significant correlations among variables and to ensure the reliability of the results, the study conducted multicollinearity diagnostics using linear regression in SPSS 29.0. All predictor variables were standardized (Z-scores) prior to analysis. The results indicated that the tolerance values of all variables ranged from 0.343 to 0.515, exceeding the reference threshold of 0.2, and the variance inflation factors (VIFs) ranged from 1.942 to 2.913, below the reference value of 5. These results suggest that multicollinearity was not a concern, and the data met the assumptions for regression analysis and chain mediation testing.

### 3.5 Regression analysis of executive function and psychological Capital in the Relationship between basketball intervention and subjective well-being

To further examine the relationships among basketball intervention, EF, PsyCap, and SWB, multiple linear regression analyses were conducted using the SPSS PROCESS macro, with 5,000 bootstrap resamples and 95% confidence intervals (Table 3). The results indicated that when basketball intervention was entered as the independent variable and SWB as the dependent variable, the regression model was significant, *F* = 72.597, *R^2^* = 0.539. Basketball intervention significantly and positively predicted SWB (*β* = 0.734, *p* < 0.001). Further analyses revealed that basketball intervention significantly and negatively predicted inhibitory control (*β* = −0.506, *p* < 0.001), working memory updating (*β* = −0.587, *p* < 0.001), and cognitive flexibility (*β* = −0.529, *p* < 0.001).

**Table 3:** Regression results for the associations among basketball intervention, executive function, psychological capital, and subjective well-being.

Regression model		Model fit indices			Regression coefficients		95% CI	
Dependent variable	Independent variable	*R*	*R* ^2^	*F*	*β*	t	Lower bound	Upper bound
SWB		0.734	0.539	72.597				
	Basketball Intervention				0.734	8.520^***^	0.562	0.907
Inhibitory Control		−0.506	0.256	21.331				
	Basketball Intervention				−0.506	−4.619^***^	−0.725	−0.287
PsyCap		0.757	0.574	41.043				
	Basketball Intervention				0.451	4.649^**^	0.257	0.644
	Inhibitory Control				−0.422	−4.356^***^	−0.616	−0.228
SWB		0.909	0.827	95.378				
	Basketball Intervention				0.289	3.984^***^	0.144	0.434
	Inhibitory Control				−0.404	−5.664^**^	−0.547	−0.261
	PsyCap				0.363	4.409^***^	0.198	0.528
Working Memory Updating		0.587	0.345	32.636				
	Basketball Intervention				−0.587	−5.713^***^	−0.793	−0.382
PsyCap		0.763	0.583	42.571				
	Basketball Intervention				0.391	3.823^**^	0.187	0.596
	Working Memory Updating				−0.465	−4.548^**^	−0.669	−0.260
SWB		0.900	0.810	85.468				
	Basketball Intervention				0.253	3.277^***^	0.099	0.408
	Working Memory Updating				−0.395	−4.917^***^	−0.556	−0.234
	PsyCap				0.375	4.307^***^	0.201	0.549
Cognitive Flexibility		0.523	0.280	24.106				
	Basketball Intervention				−0.529	−4.910^***^	−0.745	−0.314
PsyCap		0.713	0.508	31.450				
	Basketball Intervention				0.503	4.753^**^	0.292	0.715
	Cognitive Flexibility				−0.304	−2.872^**^	−0.516	−0.092
SWB		0.895	0.801	80.326	0.255	3.211^**^	0.096	0.404
	Basketball Intervention							
	Cognitive Flexibility				−0.324	−4.480^***^	−0.469	−0.180
	PsyCap				0.463	5.637^***^	0.299	0.627

SWB, Subjective Well-Being; PsyCap, Psychological Capital; *p < 0.05, **p < 0.01, ***p < 0.001.

In the regression models with PsyCap as the dependent variable, basketball intervention significantly and positively predicted PsyCap (*β* = 0.391–0.503, *p* < 0.01). In contrast, inhibitory control (*β* = −0.422, *p* < 0.001), working memory updating (*β* = −0.465, *p* < 0.01), and cognitive flexibility (*β* = −0.304, *p* < 0.01) significantly and negatively predicted PsyCap. When basketball intervention, EF, and PsyCap were simultaneously entered into the regression models, both basketball intervention (*β* = 0.253–0.289, *p* < 0.01) and PsyCap (*β* = 0.363–0.463, *p* < 0.001) significantly and positively predicted SWB. In contrast, inhibitory control (*β* = −0.404, *p* < 0.01), working memory updating (*β* = −0.395, *p* < 0.001), and cognitive flexibility (*β* = −0.324, *p* < 0.001) significantly and negatively predicted SWB.

### 3.6 Serial mediation analysis

**Figure 2:**
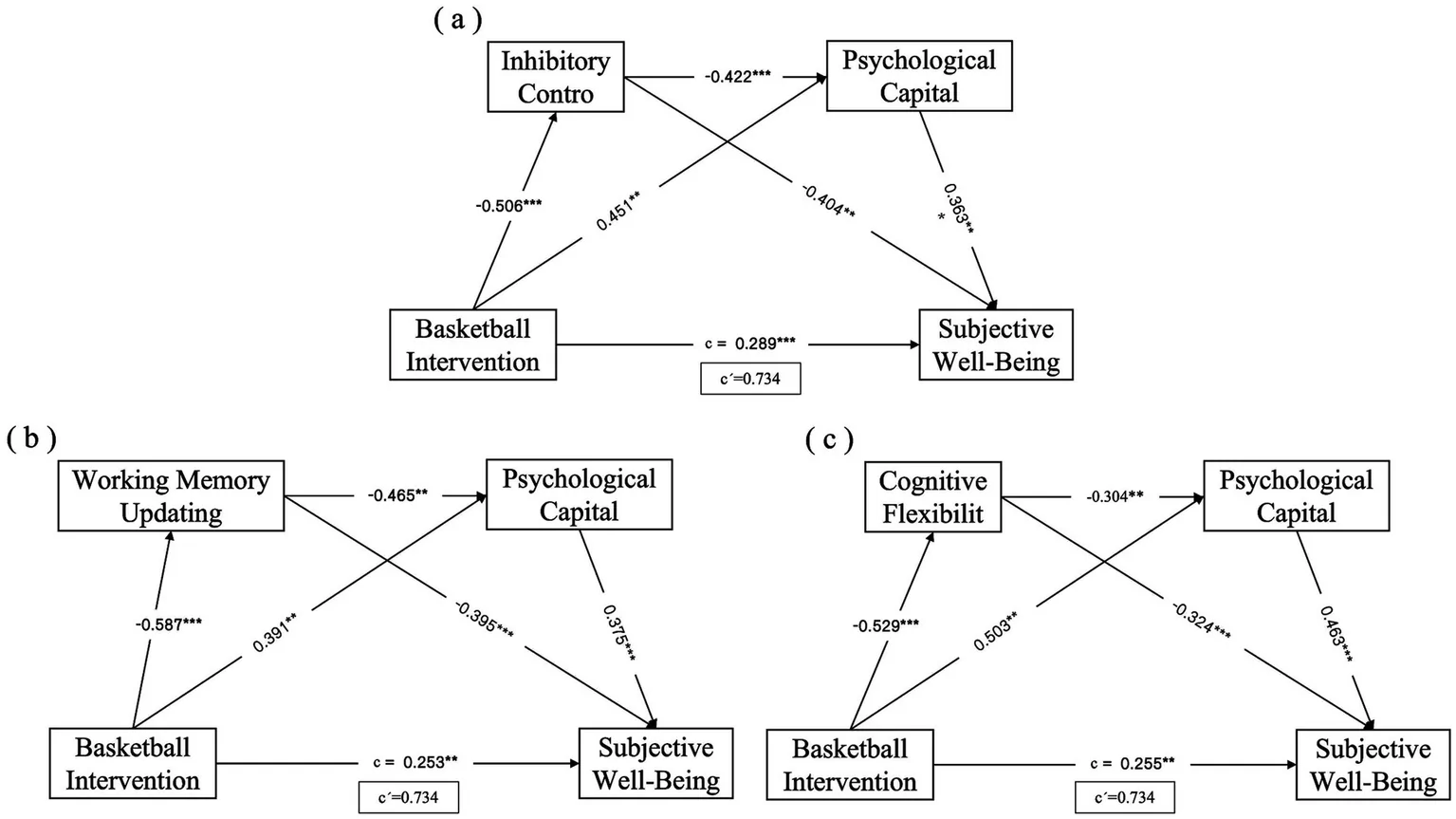
Serial mediation models of basketball intervention, executive function, psychological capital, and subjective well-being. **(a)** Serial mediation via inhibitory control and psychological capital. **(b)** Serial mediation via working memory updating and psychological capital. **(c)** Serial mediation via cognitive flexibility and psychological capital.

The results of the serial mediation analyses are presented in Table 4 and Figure 2. Overall, basketball intervention showed a significant total association with adolescents’ SWB (*β* = 0.734, 95% CI [0.562, 0.907]). After EF and PsyCap were included in the models, the direct associations remained significant (*β* = 0.253–0.289), with all 95% confidence intervals excluding zero. These findings indicate that basketball intervention showed both direct and indirect statistical associations with adolescents’ SWB through EF and PsyCap.”

**Table 4:** Serial mediation results for the indirect associations among basketball intervention, inhibitory control, psychological capital, and subjective well-being.

Effect type	Pathway	Effect	Boot *SE*	95% CI		Effect Proportion (%)
				LL	UL	
Direct effect	Basketball Intervention → SWB	0.289	0.073	0.144	0.434	39.37
Total indirect effect	Basketball Intervention → SWB	0.446	0.078	0.325	0.627	60.76
Indirect effect	Basketball Intervention → Inhibitory Control → SWB	0.204	0.059	0.139	0.360	27.80
	Basketball Intervention → PsyCap → SWB	0.164	0.056	0.058	0.279	22.34
	Basketball Intervention → Inhibitory Control → PsyCap → SWB	0.078	0.030	0.028	0.145	10.63
Total effect	Basketball Intervention → SWB	0.734	0.086	0.562	0.907	
Direct effect	Basketball Intervention → SWB	0.253	0.077	0.099	0.408	34.47
Total indirect effect	Basketball Intervention → SWB	0.481	0.080	0.325	0.640	65.53
Indirect effect	Basketball Intervention → Working Memory Updating → SWB	0.232	0.060	0.120	0.354	31.61
	Basketball Intervention → PsyCap → SWB	0.147	0.046	0.063	0.244	20.03
	Basketball Intervention → Working Memory Updating → PsyCap → SWB	0.102	0.038	0.038	0.186	13.90
Total effect	Basketball Intervention → SWB	0.734	0.086	0.562	0.907	
Direct effect	Basketball Intervention → SWB	0.255	0.086	0.096	0.404	34.74
Total indirect effect	Basketball Intervention → SWB	0.479	0.079	0.325	0.636	65.26
Indirect effect	Basketball Intervention → Cognitive Flexibility → SWB	0.172	0.053	0.067	0.282	23.43
	Basketball Intervention → PsyCap → SWB	0.233	0.063	0.115	0.359	31.74
	Basketball Intervention → Cognitive Flexibility → PsyCap → SWB	0.075	0.033	0.021	0.148	10.22
Total effect	Basketball Intervention → SWB	0.734	0.086	0.562	0.907	

Further analyses showed that the total indirect associations in all three models exceeded the corresponding direct associations. Specifically, the total indirect association was 0.446 in the inhibitory control model, accounting for 60.76% of the total association; 0.481 in the working memory updating model, accounting for 65.53% of the total association; and 0.479 in the cognitive flexibility model, accounting for 65.26% of the total association. These results suggest that the observed associations between basketball intervention and SWB were primarily accounted for by the indirect pathways involving EF and PsyCap.

For the specific mediation pathways, basketball intervention showed significant indirect associations with SWB through inhibitory control, working memory updating, and cognitive flexibility, with effect sizes of 0.204 (95% CI [0.139, 0.360]), 0.232 (95% CI [0.120, 0.354]), and 0.172 (95% CI [0.067, 0.282]), respectively. In addition, the indirect effects through PsyCap were 0.164 (95% CI [0.058, 0.279]), 0.147 (95% CI [0.063, 0.244]), and.233 (95% CI [0.115, 0.359]), respectively, all of which reached statistical significance.

More importantly, all three dimensions of EF formed significant serial mediation pathways through PsyCap. Specifically, the serial mediation effect of the pathway “Basketball Intervention → Inhibitory Control → Psychological Capital → Subjective Well-Being” was 0.078, accounting for 10.63% of the total effect. The corresponding serial mediation effects were 0.102 (13.90% of the total effect) for the pathway “Basketball Intervention → Working Memory Updating → Psychological Capital → Subjective Well-Being” and 0.075 (10.22% of the total effect) for the pathway “Basketball Intervention → Cognitive Flexibility → Psychological Capital → Subjective Well-Being.” In all cases, the 95% confidence intervals did not include zero.

## 4 Discussion

This study systematically examined the associations of basketball intervention with adolescents’ SWB and the underlying mechanisms through which these associations occur. The findings demonstrated that basketball intervention was significantly associated with higher adolescents’ SWB, with EF and PsyCap jointly serving as significant serial indirect pathways. Specifically, basketball intervention influenced SWB through improvements in inhibitory control and working memory updating, with a marginal indirect effect through cognitive flexibility, but also exerted additional indirect effects through the enhancement of PsyCap. By integrating EF and PsyCap into a unified analytical framework, the present study further revealed a synergistic pathway through which cognitive regulatory capacities and positive psychological resources contribute to the development of SWB. These findings provide new evidence for understanding the mechanisms underlying the associations of sport-based interventions with adolescents’ well-being.

### 4.1 Direct effect of basketball intervention on adolescents’ subjective well-being

The findings of the present study demonstrated that basketball intervention was significantly associated with higher adolescents’ SWB, supporting Hypothesis 1 and aligning with previous evidence indicating that exercise is associated with SWB (Fu et al., 2025). Previous studies have shown that regular physical activity is associated with better mental health, higher life satisfaction, and greater adolescents’ SWB (Ruiz-Ranz and Asín-Izquierdo, 2025). However, most existing studies have focused on overall physical activity levels and have primarily relied on physical activity questionnaires to examine the association between physical activity and SWB. Relatively limited attention was given to the actual benefits of structured team-sport interventions (Fuentealba-Urra et al., 2023). In contrast, the present study implemented a sustained basketball intervention as a representative form of team sport, providing further evidence from an experimental design perspective for the positive associations between exercise participation and adolescents’ SWB.

The present study found that significantly higher scores were observed in all dimensions of SWB except family satisfaction and academic satisfaction, including school satisfaction, environmental satisfaction, freedom satisfaction, and friendship satisfaction. This finding suggests that the associations of exercise intervention may vary across different dimensions of SWB (Mistry et al., 2024). One possible explanation is that the intervention was conducted primarily within the school context and was characterized by peer interaction, teamwork, and the pursuit of shared goals. As a result, it may have been more strongly associated with well-being experiences related to school life and social interaction, while showing weaker associations with relatively stable life domains, such as family relationships and academic pressure.

In addition, the present study found that basketball intervention showed differential associations across various dimensions of SWB. The observed differences may be partly attributable to the multidimensional nature of the measurement instrument used in the present study. Unlike the Satisfaction with Life Scale (SWLS; Diener et al., 1985) and the Positive and Negative Affect Schedule (PANAS; Watson et al., 1988), which primarily assess overall levels of well-being, the Adolescent SWB Scale used in the present study incorporates dimensions of family, school, friendship, environmental, and freedom satisfaction. This multidimensional framework enables a more comprehensive assessment of the structural characteristics of SWB among Chinese adolescents within their real-life developmental contexts (Zhang et al., 2025). Therefore, the present study not only confirmed the positive effect of basketball participation on adolescents’ SWB but also revealed differential sensitivities of specific well-being dimensions to exercise intervention. These findings provide evidence from an experimental design perspective for the positive association between exercise participation and adolescents’ SWB.

### 4.2 The mediating role of executive function in the relationship between basketball intervention and adolescents’ subjective well-being

The results of the present study demonstrated that EF showed a significant indirect association in the relationship between basketball intervention and adolescents’ SWB, thereby supporting Hypothesis 2. This finding extends previous research on the relationship between physical activity and SWB and provides new empirical evidence for understanding the cognitive mechanisms through which exercise intervention is associated with adolescents’ SWB.

The mediating pathway involving EF observed in the present study may be attributable to the open-skill characteristics inherent in basketball (Lai et al., 2024; Iván-Baragaño et al., 2025). Meanwhile, higher levels of EF enable individuals to regulate emotions more effectively, cope with stress more adaptively, and manage goals more efficiently. These advantages may, in turn, enhance positive emotional experiences and promote SWB (Rodas et al., 2024). Therefore, the present findings suggest that basketball intervention is associated with the development of EF, which may be further linked to higher SWB.

However, it should be noted that the mediating effect of EF identified in the present study may, to some extent, be influenced by the characteristics of the sport itself. Compared with closed-skill sports such as athletics and weightlifting, basketball is characterized by greater environmental variability and higher cognitive engagement demands (Koch and Krenn, 2021). These characteristics may represent one important reason why basketball is more likely to facilitate the development of EF. Therefore, the mediating role of EF observed in the present study may not be equally pronounced in exercise modes characterized by lower cognitive demands.

Regarding the three subcomponents of EF the mediating effects observed in the present study were not equally pronounced. Specifically, the indirect pathways through inhibitory control, working memory updating, and cognitive flexibility were all statistically significant. However, the intervention effect on cognitive flexibility was only marginally significant (*p* = 0.047). Moreover, its mediating pathway was relatively weaker compared to the other two components. This pattern suggests that cognitive flexibility may play a supplementary rather than a central role in the cognitive pathway linking basketball intervention to adolescents’ SWB. One possible explanation is that basketball, as an open-skill sport, places greater demands on rapid inhibition of irrelevant responses and continuous updating of working memory. In contrast, the demands on cognitive flexibility, though present, may be less intensive during typical gameplay scenarios. Future research should further examine whether different types of basketball training protocols (tactical drills vs. open play) differentially engage cognitive flexibility.

### 4.3 The mediating role of psychological Capital in the Relationship between Basketball Intervention and Adolescents’ subjective well-being

The present study found that PsyCap showed a significant indirect association in the relationship between basketball intervention and adolescents’ SWB, thereby supporting Hypothesis 3. Unlike previous studies, the present study revealed an underlying psychological mechanism through which basketball intervention is associated with adolescents’ SWB. The findings further suggest that higher SWB is an important positive psychological correlate of basketball intervention, thereby extending existing explanations regarding the mechanisms linking exercise intervention and SWB.

Notably, unlike previous studies that primarily focused on positive emotional experiences or positive psychological states (Yıldırım et al., 2025), the present study specifically examined PsyCap as a positive psychological resource. The four core dimensions of p PsyCap (self-efficacy, hope, optimism, and resilience) may provide a more comprehensive reflection of adolescents’ psychological development than positive emotional experiences alone, particularly in terms of goal persistence, stress coping, self-beliefs, and positive cognition (Tang et al., 2025). Furthermore, the positive effect of basketball intervention on PsyCap may, to some extent, be influenced by the distinctive characteristics of team sports. Compared with individual sports, team sports typically involve greater interpersonal interaction, social support, and cooperative experiences, all of which may be more conducive to the development of positive psychological resources (Wade et al., 2026). Higher levels of PsyCap enable individuals to respond more positively to stress and challenges and to maintain higher levels of SWB in difficult situations (Zhou et al., 2025). Therefore, basketball intervention may be associated with higher adolescents’ SWB through its association with PsyCap.

### 4.4 Serial mediation analysis of executive function and psychological capital

The present study found that basketball intervention showed positive indirect associations with adolescents’ SWB through the serial pathway of EF and PsyCap. Specifically, higher levels of basketball intervention were related to higher EF, which in turn was positively correlated with higher PsyCap, and this was further associated with higher SWB. These findings provide support for Hypothesis 4.

Previous studies have demonstrated that physical activity can promote EF and enhance PsyCap (Johansson et al., 2022). However, the present study simultaneously examined the sequential pathway linking EF and PsyCap within a unified analytical framework. Higher levels of EF may provide important cognitive support for the development of PsyCap (Kattner, 2021). Specifically, effective inhibitory control, working memory updating, and cognitive flexibility enable individuals to regulate negative information, maintain goal-directed behaviors, and adapt to changing environmental demands (Nasreen et al., 2024). These cognitive advantages may facilitate the development of self-efficacy, optimism, hope, and resilience, which constitute the core components of PsyCap (Daume et al., 2024). This finding moves beyond explanations that rely on a single psychological mechanism to account for the effects of exercise on SWB. Moreover, by integrating cognitive regulatory capacity and positive psychological resources into a unified framework, the present study further elucidated the underlying psychological mechanism through which basketball intervention promotes adolescents’ SWB.

One possible explanation for the observed serial mediation effect is the synergistic interaction between cognitive regulatory capacity and emotion regulation processes. Previous research has shown that individuals with higher levels of EF typically possess stronger cognitive control abilities, enabling them to regulate emotions effectively, reappraise situational information, and maintain positive psychological states. These cognitive advantages may provide an important foundation for the development of PsyCap (Halse et al., 2024). Furthermore, research has demonstrated that EF plays a critical role in regulating psychological processes and facilitating adaptive responses in dynamic environments (Rompilla Jr et al., 2025).

From the perspective of embodied cognition theory, basketball is not merely a form of physical activity but also a complex behavioral activity that requires substantial cognitive engagement (Ge et al., 2021). During sports participation, individuals are required to continuously engage in decision-making, attentional shifting, and behavioral inhibition. These cognitively demanding activities may facilitate the development of EF (Madinabeitia-Cabrera et al., 2023). Improvements in EF may, in turn, enhance goal-directed behavior and adaptive coping strategies. As these cognitive capacities increase, individuals may be more likely to adopt positive attributional styles, thereby fostering the development of key dimensions of PsyCap, such as optimism and resilience (Koay and Meter, 2023). These positive psychological resources may subsequently contribute to enhanced SWB.

Finally, it should be acknowledged that the present study assessed only psychological variables. Therefore, the explanations regarding the neural regions and physiological mechanisms underlying the observed serial mediation pathway remain speculative and are primarily based on inferences drawn from existing literature. In addition, the present study was conducted in China, and the generalizability of the findings to other populations remains uncertain. Nevertheless, there is currently insufficient evidence to suggest that the observed relationships are unique to Chinese adolescents.

## 5 Limitations and future directions

This study provides theoretical contributions to the empirical evidence regarding the relationship among basketball intervention, EF, PsyCap, and SWB, but it still has the following limitations: First, the sample group is relatively homogeneous and limited in representativeness, and therefore the generalization of the findings to other populations should be treated with caution. Additionally, the sample size (*N* = 64) is relatively modest. However, simulation research suggests that for mediation models with moderate-to-large effect sizes, a sample of 50–100 participants can achieve statistical power above 0.80 when using the bootstrap method. Given that the total effect (*β* = 0.734) and the indirect effect (0.479) in this study were moderate to large, the sample size was considered adequate for detecting the hypothesized mediation effects. Nevertheless, future studies with larger and more diverse samples would further enhance the robustness and generalizability of the findings. Second, this study only examined short-term intervention effects and did not explore the long-term effects of basketball intervention; future studies may use longitudinal designs to investigate the long-term impact of exercise interventions on adolescents’ psychological development. Third, the measurement dimensions in this study were relatively limited, and the discriminant validity between SWB and PsyCap requires further examination. The confirmatory factor analysis results (CFA) (see Supplementary Table S5) revealed a high latent correlation between SWB and PsyCap suggesting substantial empirical overlap between the two constructs in the current adolescent sample. This result may be attributable to the relatively small sample size, which could lead to estimation instability and the Heywood case observed in the CFA. Nevertheless, multicollinearity diagnostics indicated that this high correlation did not compromise the subsequent mediation analyses, and the core findings remained robust. Future research should employ larger sample sizes or multi-source data to further examine the discriminant validity of these two constructs and improve the precision of mediation effect estimation. Fourth, the refinement of intervention protocols. This study did not thoroughly examine the moderating effects of different exercise prescription parameters (intensity, complexity, frequency) and individual characteristics (gender, baseline fitness) on the mechanism pathways. Fifth, another limitation concerns the control condition. Because the control group participated only in regular physical education classes, it remains difficult to determine whether the observed benefits are specific to basketball itself or whether they reflect more general effects of physical activity, social interaction, or team participation. The inclusion of an active control group (other team sports or structured exercise programs) in future studies would help clarify the unique contributions of the basketball intervention and allow for a more precise interpretation of the intervention effects. Sixth, EF, PsyCap, and SWB were measured at the same post-intervention time point. Although the randomized design supports the direction of the proposed pathways, this simultaneous measurement limits the ability to fully establish temporal precedence between EF and PsyCap. Therefore, the sequential pathway should be interpreted as statistical indirect associations consistent with the proposed model, rather than as definitive evidence of a temporal or causal sequence. Future studies with multiple follow-up assessments are needed to further validate the temporal dynamics of these processes. Seventh, the present study did not include baseline scores of the mediators and outcome as covariates in the serial mediation model. Without baseline adjustment, the estimated indirect effects may reflect pre-existing relationships among variables rather than true intervention-induced changes. Methodological literature has recommended that baseline measurements of intermediate and outcome variables be included in mediation analyses of intervention studies to control for confounding bias (Landau et al., 2018; Loh and Ren, 2023). Future research adopting longitudinal designs with multiple follow-up time points and baseline covariates is needed to validate the present findings.

## 6 Conclusion

The findings of the present study indicate that basketball intervention was significantly associated with higher adolescents’ SWB. The associations of basketball intervention were reflected not only in its direct association with SWB but also through multiple indirect pathways involving EF and PsyCap. In particular, the serial pathway of “basketball intervention → executive function → psychological capital → subjective well-being” highlights the interconnected roles of cognitive abilities and psychological resources in promoting adolescents’ well-being. Furthermore, the findings provide empirical evidence for understanding the cognitive–emotional mechanisms through which basketball intervention promotes adolescent mental health.

## Data Availability

The original contributions presented in the study are included in the article/Supplementary material, further inquiries can be directed to the corresponding author.
