# Supplementary material for: From the court to the heart: basketball intervention is associated with adolescents’ subjective well-being—a serial mediation analysis of executive function and psychological capital
Source: Front Psychol. 2026 Aug 10;17:1929704. doi: 10.3389/fpsyg.2026.1929704 (PMC13500231; doi:10.3389/fpsyg.2026.1929704)
Supplement: Supplementary file 1 [file Data_Sheet_1.PDF]

Supplementary Material

1 Supplementary Methods

Assessment of Normal Cognitive Functioning

Normal cognitive functioning was operationally defined as enrollment in a regular mainstream classroom (indicating no intellectual disability or special education placement), absence of a documented diagnosis of intellectual disability or learning disorder as verified through school enrollment records, and teacher confirmation of age-appropriate learning capacity (Pesce et al., 2016). This information was obtained by the research team during the participant recruitment screening phase, through consultation with the school's academic affairs office and the homeroom teachers. Additionally, all participants completed a task comprehension check at baseline, in which they achieved above-chance accuracy (>50%) on all three executive function tasks, confirming adequate understanding of the task instructions.

Pesce, C., Masci, I., Marchetti, R., Vazou, S., Sääkslahti, A., & Tomporowski, P. D. (2016). Deliberate play and preparation jointly benefit motor and cognitive development: mediated and moderated effects. Frontiers in psychology, 7, 349.

2 Supplementary Tables

Table S1 Training Program Schedule

Week	Basketball Intervention Program
Week 1	Ball-handling drills, basic dribbling, passing techniques, and physical conditioning exercises.
Week 2	Ball-handling drills, basic dribbling, passing techniques, and physical conditioning exercises.
Week 3	Ball-handling drills, dynamic dribbling, passing and receiving techniques, and physical conditioning.
Week 4	Footwork drills, dynamic dribbling, set shooting practice, and physical conditioning.
Week 5	Footwork drills, combined dynamic dribbling, and set shooting practice.
Week 6	Footwork drills, combined dynamic dribbling, dynamic shooting (lay-ups), and defensive techniques (including defensive footwork and skills).
Week 7	Combined dynamic dribbling, defensive techniques, and offensive tactical training (half-court coordination, screens, cuts, and man-to-man defense).
Week 8	Offensive tactical training and half-court scrimmage.
Week 9	Defensive tactical training and half-court scrimmage.
Week 10	Integrated training, including 4v4 game-based practice.
Week 11	Full-court 5v5 competitive games.
Week 12	Summary and performance presentation, including skill demonstrations and mini-competitions.

Table S2 Specific Training Components

Program	Training Components
Ball-handling	Finger tapping drills, ball wrapping exercises, single- and double-hand toss-and-catch, alternating dribbling with both hands.
Passing	Two-hand chest pass, bounce pass, one-hand shoulder pass, overhead pass.
Footwork	Linear sprinting, forward and backward movement, V-shaped running, multidirectional changes (forward, backward, lateral), lateral movement, running, defensive slides, crossover steps, and jab steps.
Dynamic shooting	Lay-up shooting.
Dynamic dribbling	High–low dribbling, crossover dribbling, spin dribbling, stop-and-go dribbling.
Combined dynamic dribbling	High–low dribbling, between-the-legs dribbling, crossover with stop-and-go, spin dribbling.
Game-based practice	2v1, 3v2, half-court 3v3, half-court 4v4, full-court 5v5.
Physical conditioning	High knees, burpees, squat jumps, vertical jumps, shuttle runs, frog jumps, push-ups, sit-ups, and plank exercises.

Table S3 Detailed experimental parameters for the Flanker, 2-back, and More-odd shifting tasks used to assess inhibitory control, working memory updating, and cognitive flexibility, respectively.

Parameter	Flanker Task	2-back Task	More-odd Shifting Task
Practice trials	12 (6 congruent, 6 incongruent)	12 (6 match, 6 non-match)	12 (4 magnitude, 4 parity, 4 mixed)
Formal trials	48 (24 congruent, 24 incongruent)	25 (12–13 match, 12–13 non-match)	48 (16 magnitude, 16 parity, 16 mixed)
Stimulus characteristics	Five horizontally arranged arrows; congruent (<<<<< or >>>>>); incongruent (<<><< or >><>>)	Five uppercase letters (Q, W, E, R, T) presented in white on a black background	Digits 1–9 (excluding 5) presented in white (magnitude judgment) or green (parity judgment) on a black background
Fixation cross duration	500 ms	500 ms	500 ms
Stimulus duration	1000 ms	2000 ms	2000 ms
Interstimulus interval (ISI)	2000 ms	3000 ms	3000 ms
Response keys	"F" for left-pointing central arrow; "J" for right-pointing central arrow	"F" for match; "J" for non-match	Magnitude: "<5" press "F", ">5" press "J"; Parity: odd press "F", even press "J"; Mixed: rule determined by digit color
Trial type proportions	Congruent: 50%; Incongruent: 50%	Match: ~50%; Non-match: ~50%	Magnitude: 33.3%; Parity: 33.3%; Mixed: 33.3%

Outcome measure	Conflict effect (incongruent RT – congruent RT)	Mean RT for correct responses	Switch cost (switch trial RT – non-switch trial RT
Interpretation	Smaller values = better inhibitory control	Shorter values = better updating ability	Smaller values = better cognitive flexibility
RT exclusion criteria	RT < 200 ms or > 2000 ms (< 2% of trials)	RT < 200 ms or > 2000 ms (< 2% of trials)	RT < 200 ms or > 2000 ms (< 2% of trials)
Task order	1st	2nd	3rd
Practice-to-formal criterion	≥ 80% accuracy in practice	≥ 80% accuracy in practice	≥ 80% accuracy in practice

Table S4 Baseline Equivalence Between the Experimental and Control Groups

Variable	Dimension	Experimental Group (N=32)	Control Group （N=32）	t	P
SWB	Family Satisfaction	35.38±3.62	34.88±4.50	0.490	0.625
	Academic Satisfaction	24.44±6.55	21.53±5.412	1.934	0.058
	School Satisfaction	26.84±6.65	25.31±4.48	1.080	0.284
	Freedom Satisfaction	24.72±4.00	23.19±3.68	1.59	0.116
	Environmental Satisfaction	24.78±4.58	23.56±5.18	0.997	0.32
	Friendship Satisfaction	39.47±5.16	37.31±5.43	1.630	0.108
	Total SWB	175.22±20.07	174.91±12.07	0.075	0.940
EF	Inhibitory Control	30.01±9.93	32.20±6.56	-1.039	0.303
	Working Memory Updating	990.61±159.78	971.22±171.51	0.468	0.642

	Cognitive Flexibility	550.18±94.43	579.96±88.48	-1.302	0.198
PsyCap	Self-Efficacy	24.88±6.19	23.09±7.05	1.074	0.287
	Resilience	27.19±5.08	25.75±4.64	1.182	0.242
	Hope	24.19±4.61	23.91±4.36	0.251	0.803
	Optimism	25.66±4.85	23.97±4.40	1.458	0.150
	Total PsyCap	100.78±17.22	98.94±11.07	0.509	0.612

Notes: (1) SWB = Subjective Well-Being; (2) EF = Executive Function; (3) PsyCap = Psychological Capital

Table S5 Confirmatory Factor Analysis Results

**Model**	**χ^2**	**df**	**χ^2/df**	**CFI**	**TLI**	**RMSEA (90% CI)**	**SRMR**
Two-Factor Model (SWB &PsyCap)	52.579	34	1.55	0.933	0.911	0.092 [0.036, 0.139]	0.065
Single-factor	52.768	35	1.51	0.936	0.918	0.089 [0.031, 0.136]	0.065
