# Supplementary material for: From the court to the heart: basketball intervention is associated with adolescents’ subjective well-being—a serial mediation analysis of executive function and psychological capital
Source: Front Psychol. 2026 Aug 10;17:1929704. doi: 10.3389/fpsyg.2026.1929704 (PMC13500231; doi:10.3389/fpsyg.2026.1929704)
Supplement: Supplementary file 2 [file Table_1.DOCX]

Supplementary Material

# 1 Supplementary Methods

Assessment of Normal Cognitive Functioning

Normal cognitive functioning was operationally defined as enrollment in a regular mainstream classroom (indicating no intellectual disability or special education placement), absence of a documented diagnosis of intellectual disability or learning disorder as verified through school enrollment records, and teacher confirmation of age-appropriate learning capacity (Pesce et al., 2016). This information was obtained by the research team during the participant recruitment screening phase, through consultation with the school’s academic affairs office and the homeroom teachers. Additionally, all participants completed a task comprehension check at baseline, in which they achieved above-chance accuracy (>50%) on all three executive function tasks, confirming adequate understanding of the task instructions.

Pesce, C., Masci, I., Marchetti, R., Vazou, S., Sääkslahti, A., & Tomporowski, P. D. (2016). Deliberate play and preparation jointly benefit motor and cognitive development: mediated and moderated effects. Frontiers in psychology, 7, 349.

# 2 Supplementary Tables

Table S1 Training Program Schedule

| Week | Basketball Intervention Program |
| --- | --- |
| Week 1 | Ball-handling drills, basic dribbling, passing techniques, and physical conditioning exercises. |
| Week 2 | Ball-handling drills, basic dribbling, passing techniques, and physical conditioning exercises. |
| Week 3 | Ball-handling drills, dynamic dribbling, passing and receiving techniques, and physical conditioning. |
| Week 4 | Footwork drills, dynamic dribbling, set shooting practice, and physical conditioning. |
| Week 5 | Footwork drills, combined dynamic dribbling, and set shooting practice. |
| Week 6 | Footwork drills, combined dynamic dribbling, dynamic shooting (lay-ups), and defensive techniques (including defensive footwork and skills). |
| Week 7 | Combined dynamic dribbling, defensive techniques, and offensive tactical training (half-court coordination, screens, cuts, and man-to-man defense). |
| Week 8 | Offensive tactical training and half-court scrimmage. |
| Week 9 | Defensive tactical training and half-court scrimmage. |
| Week 10 | Integrated training, including 4v4 game-based practice. |
| Week 11 | Full-court 5v5 competitive games. |
| Week 12 | Summary and performance presentation, including skill demonstrations and mini-competitions. |

Table S2 Specific Training Components

| Program | Training Components |
| --- | --- |
| **Ball-handling** | Finger tapping drills, ball wrapping exercises, single- and double-hand toss-and-catch, alternating dribbling with both hands. |
| **Passing** | Two-hand chest pass, bounce pass, one-hand shoulder pass, overhead pass. |
| **Footwork** | Linear sprinting, forward and backward movement, V-shaped running, multidirectional changes (forward, backward, lateral), lateral movement, running, defensive slides, crossover steps, and jab steps. |
| **Dynamic shooting** | Lay-up shooting. |
| **Dynamic dribbling** | High–low dribbling, crossover dribbling, spin dribbling, stop-and-go dribbling. |
| **Combined dynamic dribbling** | High–low dribbling, between-the-legs dribbling, crossover with stop-and-go, spin dribbling. |
| **Game-based practice** | 2v1, 3v2, half-court 3v3, half-court 4v4, full-court 5v5. |
| **Physical conditioning** | High knees, burpees, squat jumps, vertical jumps, shuttle runs, frog jumps, push-ups, sit-ups, and plank exercises. |

Table S3 Detailed experimental parameters for the Flanker, 2-back, and More-odd shifting tasks used to assess inhibitory control, working memory updating, and cognitive flexibility, respectively.

| **Parameter** | **Flanker Task** | **2-back Task** | **More-odd Shifting Task** |
| --- | --- | --- | --- |
| Practice trials | 12 (6 congruent, 6 incongruent) | 12 (6 match, 6 non-match) | 12 (4 magnitude, 4 parity, 4 mixed) |
| Formal trials | 48 (24 congruent, 24 incongruent) | 25 (12–13 match, 12–13 non-match) | 48 (16 magnitude, 16 parity, 16 mixed) |
| Stimulus characteristics | Five horizontally arranged arrows; congruent (<<<<< or >>>>>); incongruent (<<><< or >><>>) | Five uppercase letters (Q, W, E, R, T) presented in white on a black background | Digits 1–9 (excluding 5) presented in white (magnitude judgment) or green (parity judgment) on a black background |
| Fixation cross duration | 500 ms | 500 ms | 500 ms |
| Stimulus duration | 1000 ms | 2000 ms | 2000 ms |
| Interstimulus interval (ISI) | 2000 ms | 3000 ms | 3000 ms |
| Response keys | "F" for left-pointing central arrow; "J" for right-pointing central arrow | "F" for match; "J" for non-match | Magnitude: "<5" press "F", ">5" press "J"; Parity: odd press "F", even press "J"; Mixed: rule determined by digit color |
| Trial type proportions | Congruent: 50%; Incongruent: 50% | Match: ~50%; Non-match: ~50% | Magnitude: 33.3%; Parity: 33.3%; Mixed: 33.3% |
| Outcome measure | Conflict effect (incongruent RT – congruent RT) | Mean RT for correct responses | Switch cost (switch trial RT – non-switch trial RT |
| Interpretation | Smaller values = better inhibitory control | Shorter values = better updating ability | Smaller values = better cognitive flexibility |
| RT exclusion criteria | RT < 200 ms or > 2000 ms (< 2% of trials) | RT < 200 ms or > 2000 ms (< 2% of trials) | RT < 200 ms or > 2000 ms (< 2% of trials) |
| Task order | 1st | 2nd | 3rd |
| Practice-to-formal criterion | ≥ 80% accuracy in practice | ≥ 80% accuracy in practice | ≥ 80% accuracy in practice |

Table S4 Baseline Equivalence Between the Experimental and Control Groups

| Variable | Dimension | Experimental Group (N=32) | Control Group（N=32） | t | P |
| --- | --- | --- | --- | --- | --- |
| SWB | Family Satisfaction | 35.38±3.62 | 34.88±4.50 | 0.490 | 0.625 |
|  | Academic Satisfaction | 24.44±6.55 | 21.53±5.412 | 1.934 | 0.058 |
|  | School Satisfaction | 26.84±6.65 | 25.31±4.48 | 1.080 | 0.284 |
|  | Freedom Satisfaction | 24.72±4.00 | 23.19±3.68 | 1.59 | 0.116 |
|  | Environmental Satisfaction | 24.78±4.58 | 23.56±5.18 | 0.997 | 0.32 |
|  | Friendship Satisfaction | 39.47±5.16 | 37.31±5.43 | 1.630 | 0.108 |
|  | Total SWB | 175.22±20.07 | 174.91±12.07 | 0.075 | 0.940 |
| EF | Inhibitory Control | 30.01±9.93 | 32.20±6.56 | -1.039 | 0.303 |
|  | Working Memory Updating | 990.61±159.78 | 971.22±171.51 | 0.468 | 0.642 |
|  | Cognitive Flexibility | 550.18±94.43 | 579.96±88.48 | -1.302 | 0.198 |
| PsyCap | Self-Efficacy | 24.88±6.19 | 23.09±7.05 | 1.074 | 0.287 |
|  | Resilience | 27.19±5.08 | 25.75±4.64 | 1.182 | 0.242 |
|  | Hope | 24.19±4.61 | 23.91±4.36 | 0.251 | 0.803 |
|  | Optimism | 25.66±4.85 | 23.97±4.40 | 1.458 | 0.150 |
|  | Total PsyCap | 100.78±17.22 | 98.94±11.07 | 0.509 | 0.612 |

Notes: (1) SWB = Subjective Well-Being; (2) EF = Executive Function; (3) PsyCap = Psychological Capital

Table S5 Confirmatory Factor Analysis Results

| **Model** | **χ²** | **df** | **χ²/df** | **CFI** | **TLI** | **RMSEA** **(90% CI)** | **SRMR** |
| --- | --- | --- | --- | --- | --- | --- | --- |
| Two-Factor Model (SWB &PsyCap) | 52.579 | 34 | 1.55 | 0.933 | 0.911 | 0.092 [0.036, 0.139] | 0.065 |
| Single-factor | 52.768 | 35 | 1.51 | 0.936 | 0.918 | 0.089 [0.031, 0.136] | 0.065 |
